# Assessment of the reproducibility of bacterial membrane vesicle isolation and characterization

**DOI:** 10.20517/evcna.2025.71

**Published:** 2025-11-10

**Authors:** Jari Verbunt, Johan Jocken, Emanuel Canfora, David Barnett, Ellen E. Blaak, Paul Savelkoul, Frank Stassen

**Affiliations:** ^1^Department of Medical Microbiology, Infectious Diseases & Infection Prevention, School of Nutrition and Translational Research in Metabolism (NUTRIM), Maastricht University Medical Center+, Maastricht 6202 AZ, The Netherlands.; ^2^Department of Human Biology, School of Nutrition and Translational Research in Metabolism (NUTRIM), Maastricht University Medical Center+, Maastricht 6202 AZ, The Netherlands.

**Keywords:** Bacterial membrane vesicles, bacteria, vesicles, microbiome, microbiota, 16S rDNA sequencing, reproducibility

## Abstract

**Aim:** This study aimed to evaluate the reproducibility of the isolation and characterization of feces-derived bacterial membrane vesicles.

**Methods:** Human fecal samples (*n* = 12) stored at -80 °C were thawed, sampled, and then refrozen. From these samples, bacterial membrane vesicles were isolated through ultrafiltration, ultracentrifugation and size exclusion chromatography. Vesicle-associated DNA was characterized by marker [16 ribosomal DNA (rDNA)] sequencing to determine composition. The same fecal samples were thawed again after > 6 months of storage at -80 °C to repeat this procedure. Compositions and other vesicle characteristics were compared to investigate effects of storage and freeze/thawing on sample stability. In addition, for four of the fecal aliquots, the bacteria were subjected to marker gene sequencing alongside their derived membrane vesicles.

**Results:** No significant differences were observed in the pre- and post freeze/thawing composition of feces-derived bacterial membrane vesicles [permutational multivariate analysis of variance (PERMANOVA) *P* = 0.356] or bacteria (PERMANOVA *P* = 0.721) as determined by 16S rDNA sequencing. Additionally, no significant differences were observed in vesicle size, concentration, and associated protein or DNA content. These results indicate that, long-term storage of feces at -80 °C and an additional freeze/thawing cycle does not induce compositional or qualitative changes to vesicle repertoires.

**Conclusion:** These reproducibility findings hold great relevance for research on (gut)bacteria derived membrane vesicles. Our results indicate that fecal samples can be stably preserved at -80 °C for bacterial and vesicle isolations as their characteristics remain stable over time.

## INTRODUCTION

The intestinal microbiota are constantly interacting with each other and with the host^[1,2]^. To help predict functionality, the intestinal bacterial microbiome can be characterized through marker gene inferred compositional analyses. Such methods yield relevant information for studying diseases characterized by a divergent microbial composition. In these conditions, the outgrowth of certain bacterial taxa may produce detrimental metabolites that affect the host^[3-6]^. Sampling fecal matter provides an easy and noninvasive way to facilitate analysis of intestinal microbiota composition. This is commonly performed to study etiological roles of the gut microbiome in diseases such as obesity^[7,8]^, inflammatory bowel disease^[6]^, type 2 diabetes^[8,9]^ and depression^[5]^.

Bacterial membrane vesicles (bMVs) have gained attention for their stable nature, interactions with the host, and ability to shuttle bacterial products^[10-13]^. These vesicles carry a diverse range of cargo, including bacterial toxins, nucleic acids, lipids and metabolites derived from their producer cells^[14]^. The methods developed for bacterial composition analysis can also be used in vesicle composition analysis, as was previously demonstrated^[15]^. bMVs can be separated from parent bacteria by separation techniques based on physicochemical properties^[16]^. As biological agents, the immunological effects of bMVs on the host could be predicted by identifying their bacterial origin^[15]^. Herein, marker gene sequencing such as 16S ribosomal DNA (16S rDNA) sequencing can help elucidate the bacterial producers of bMVs, providing a basis for hypothesis testing in relating composition to functionality. Ensuring the compositional stability of gut bacteria in frozen fecal samples is crucial for reliable analyses across studies. Although previous research suggests that sample characteristics reliably remain stable at room temperature (RT) for a duration of 24 h^[17,18]^, the stability of bMV-DNA compositions in frozen fecal samples remains unexplored. Disproportionate degradation of taxon-specific 16S rDNA over time could introduce compositional changes, which could lead to poor reproducibility of results and incorrect insights into how microbial communities relate to health.

**Figure 1:**
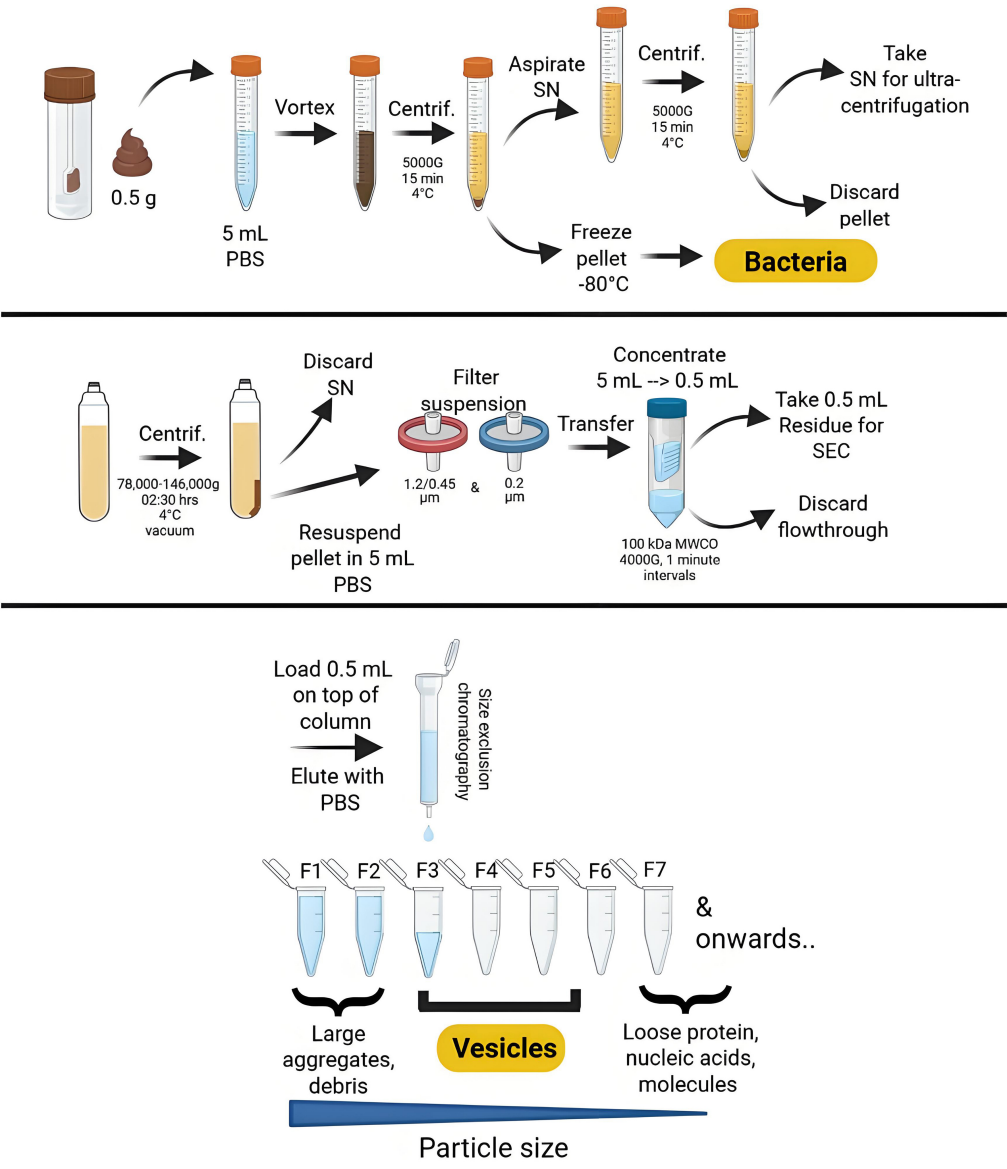
Overview of the isolation purification procedure for bacterial membrane vesicles through consecutive (ultra)centrifugation and (ultra)filtration steps, followed by size exclusion chromatography. Created using Biorender.com. PBS: Phosphate-buffered saline; SN:supernatant; SEC: size exclusion chromatography; MWCO: molecular weight cut-off; F: fraction; G: gravity (relative centrifugal force).

To address this query, we characterized the vesicle repertoire derived from 12 different fecal samples following extensive purification [Figure 1], alongside the bacterial composition of four of the samples. The bacterial and vesicle compositions were compared using 16S rDNA marker gene sequencing before and after > 6 months of fecal sample storage at -80 °C. In addition, the physical and biochemical characteristics of the vesicles were assessed with respect to size, concentration, protein, and DNA content.

## METHODS

### Fecal samples

Biobanked fecal samples stored at -80 °C were selected based on availability. According to firmly established procedures, samples were previously collected by study participants at home in 2016/2017 (and promptly frozen at -20 °C) as part of a study investigating the effects of dietary fiber intake on host metabolic health (Clinical trial No. NCT03711383). Sample donors (*N* = 12) had a mean age of 54.0 ± 11.7 years and a mean BMI of 25.5 ± 2.7 kg/m^2^. All participants were Caucasian males who were weight-stable for at least 3 months prior to the start of the study. Exclusion criteria included a diagnosis of type 2 diabetes mellitus; a history of abdominal surgery; gastrointestinal or cardiovascular diseases; hepatic or renal dysfunction; a life expectancy of less than five years; adherence to a hypocaloric diet; or the use of antibiotics, prebiotics, or probiotics during the study or within the three months prior to participation. Participants were also excluded if they were taking lipid- or glucose-lowering medications, antioxidants, β-blockers, or corticosteroids.

Written informed consent was obtained from all participants.

### Isolation of bacteria and their membrane vesicles from feces

Feces-derived bacteria and bMVs were isolated using methods previously described^[15]^. In brief, 0.5 g of feces was resuspended in 5 mL of phosphate-buffered saline (PBS, Gibco, Netherlands) to facilitate bacterial pelleting using centrifugation at 5,000 × *g* for 15 min at 4 °C. Pellets containing the bacteria were stored at -80 °C until further downstream purification. Vesicles in the supernatant were pelleted by ultracentrifugation at 78,000-146,000 for 2.5 h at 4 °C. After resuspending the vesicle pellet in PBS, vesicles were subsequently filtered through a 0.45 µm and a 0.20 µm syringe filter (Acrodisc, USA). Then, vesicle suspensions were concentrated to a total volume of 500 µL using a 100 kDa molecular weight cut-off (MWCO) ultrafilter (Amicon, USA) to allow for size exclusion chromatography (SEC). For SEC, 500 µL of vesicle concentrate was loaded on top of a 35 nm qEV original Sepharose SEC column (Izon, Netherlands). Elution was performed using PBS, and twelve 1 mL fractions were collected. Fractions 3, 4, and 5, previously identified as being enriched for bMVs^[15]^, were pooled and subjected to bMV-DNA extraction. All laboratory steps between iterations were performed by the same operator using identical equipment settings.

### DNA extraction from bacteria and bMVs

Bacterial pellets were subjected to fecal DNA extraction using 0.1 mm zirconia bead beating (Biospec) for a duration of 180 s at 5.5 m/sec. Subsequent column purification of bacterial DNA was performed using the QIAamp DNA mini kit (QIAGEN, Netherlands) according to the manufacturer’s instructions. Purified bMVs were subjected to marker gene amplification directly, without additional lysis or purification steps.

### 16S rRNA gene amplification and sequencing

For both sample types, 16S rRNA variable region amplification followed by Illumina MiSeq sequencing was conducted. The V4 region of the 16S rRNA gene was amplified using primers 515Fw (5’-GTGCCAGCMGCCGCGGTAA-3’) and 806Rv (5’-GGACTACHVGGGTWTCTAAT^*^-3’) as described previously^[19]^. Primers were synthesized by Sigma-Aldrich (Netherlands). Each polymerase chain reaction (PCR) contained 1 µL of each primer (10 pmol/µL), 0.2 µL of AccuPrime High Fidelity polymerase (Thermo Fischer Scientific), and 5 µL of AccuPrime buffer II, along with 1 µL of template DNA and 41.8 µL of molecular-grade water. The PCR protocol included an initial denaturation at 94 °C for 3 min, followed by 35 cycles (30 s at 94 °C, 45 s at 50 °C, and 60 s at 72 °C) for bMV DNA templates and 25 cycles for bacterial DNA templates. A final extension at 72 °C for 10 min completed the reaction. Quality and quantity of the amplicon libraries were assessed using a 1% agarose gel. Amplicon libraries were then purified using a Zephyr® G3 next-generation sequencing (NGS) Workstation (PerkinElmer, Waltham, MA, USA) and Agencourt AMPure beads (Brea, USA). Quantification of the amplified DNA was done using PicoGreen Quant-iT (Invitrogen), after which amplicons were pooled in equimolar concentrations to achieve a final DNA concentration of 1 ng/µL. Sequencing was performed on the Illumina MiSeq system using the MiSeq V3 reagent kit (2 × 250 cycles, Illumina).

### Nanoparticle tracking analysis

Concentrations and sizes of purified bMVs were determined by nanoparticle tracking analysis (NTA, Zetaview, Particle Metrix) employing a 488 nm laser. Vesicles were diluted by a factor of 500 in PBS (Gibco) prior to measurements.

### bMV protein quantification

Protein content in purified vesicle fractions was quantified using a modified Lowry protein quantification procedure (Micro Lowry with Peterson’s modification, Sigma) with a protein precipitation step for improved sensitivity^[20]^. In brief, to each vesicle sample, 12.5 μL of deoxycholate solution was added and incubated for 10 min at RT. Subsequently, 12.5 μL of trichloroacetic acid was added, followed by thorough mixing and sample centrifugation for 5 min, > 11,000 × *g* at RT. Supernatant was discarded and samples were diluted with 62.5 μL of distilled water and 62.5 μL of Lowry reagent following RT incubation for 20 min. Lastly, 62.5 μL of Folin phenol reagent was added and samples were incubated at RT for 30 min in the dark. Absorption was measured at 700 nm using a Spectramax ID3 plate reader (San Jose, CA).

### Qbit DNA quantification of bMVs

The DNA content of bMVs was quantified using a Qubit High Sensitivity dsDNA Assay (Thermo Fisher Scientific) and an accessory Qubit 3.0 fluorometer. For measurements, 10 µL of the sample was diluted in 190 µL of Qubit reagent mix.

### Cryo-TEM imaging of bMVs

Three microliters of purified vesicle suspension in PBS were deposited onto a glow-discharged holey carbon grid to leave a thin film spanning the grid holes. Samples were maintained at 95% humidity prior to plunge freezing in liquid ethane using a Vitrobot (FEI, Eindhoven, The Netherlands). Vitrified samples were transferred to a Tecnai Arctica Cryo-Transmission Electron Microscope (ThermoFisher, Eindhoven, The Netherlands). Imaging was conducted at a voltage of 200 kV.

### Data analysis

Amplicon data preprocessing for analysis was performed using Divisive Amplicon Denoising Algorithm 2 (DADA2) denoising (version 1.28.0) and taxonomic classification using SILVA 138.1.

### Statistical analysis

Statistical testing of analyzed data was performed in R version 4.2.0 using RStudio (version 2024.04.0 + 735) and the microViz package (version 0.12.1)^[21]^. Permutational multivariate analysis of variance (PERMANOVA) was executed to compare differences between groups, using Bray-Curtis distances calculated at the Genus level. Differences in bacterial richness and diversity between batch years were assessed using the Wilcoxon signed-rank test for paired samples. Paired sample t-testing was performed to assess physical and biochemical characteristics of the vesicles between time point iterations, using a significance threshold of *P* < 0.05.

## RESULTS

### Vesicle DNA compositions remain stable over time

**Figure 2:**
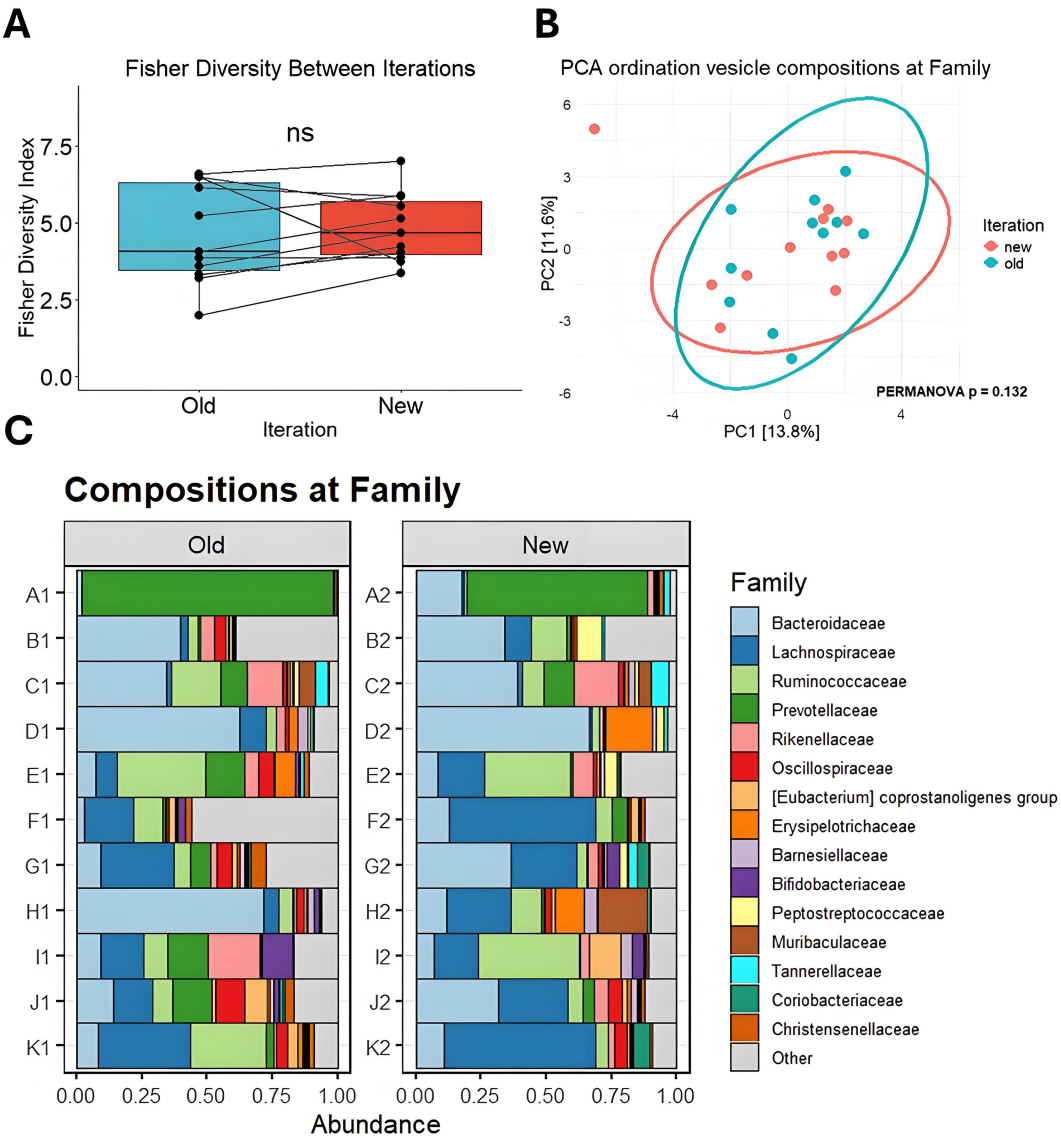
Comparison of bMV compositions between iterations. (A) Fisher’s alpha diversity at family taxonomy. Data are presented as means ± SEM; (B) Principal component analysis ordination biplot of CLR-transformed features at the taxonomic Family level. 95% confidence ellipses are drawn per iteration; (C) Bar charts showing the proportional abundance of detected taxa from vesicle DNA at the Family level between iterations. SEM: Standard error of the mean; bMV: bacterial membrane vesicle; NS: not significant; PCA: principal component analysis; CLR: centered log-ratio; PERMANOVA: permutational multivariate analysis of variance.

Analysis of bMV DNA across iterations revealed no significant compositional differences, indicating that fecal matter stored at -80 °C stably preserves bMV DNA [Figure 2]. Alpha diversity calculations indicated no significant differences in median diversity between time points, suggesting that both the number and distribution of taxa in bMVs remained stable [Figure 2A and B]. No significant compositional differences are observed at the Family (PERMANOVA *P* = 0.132) [Figure 2B] or Genus level [Supplementary Figure 1] between iterations, although some individual samples do differ more than others. In contrast, baseline differences between the vesicle compositions from different samples are substantial, with some samples dominated by Prevotellaceae and others by Bacteroidaceae [Figure 2C].

### Vesicle physicochemical characteristics remain stable over time

**Figure 3:**
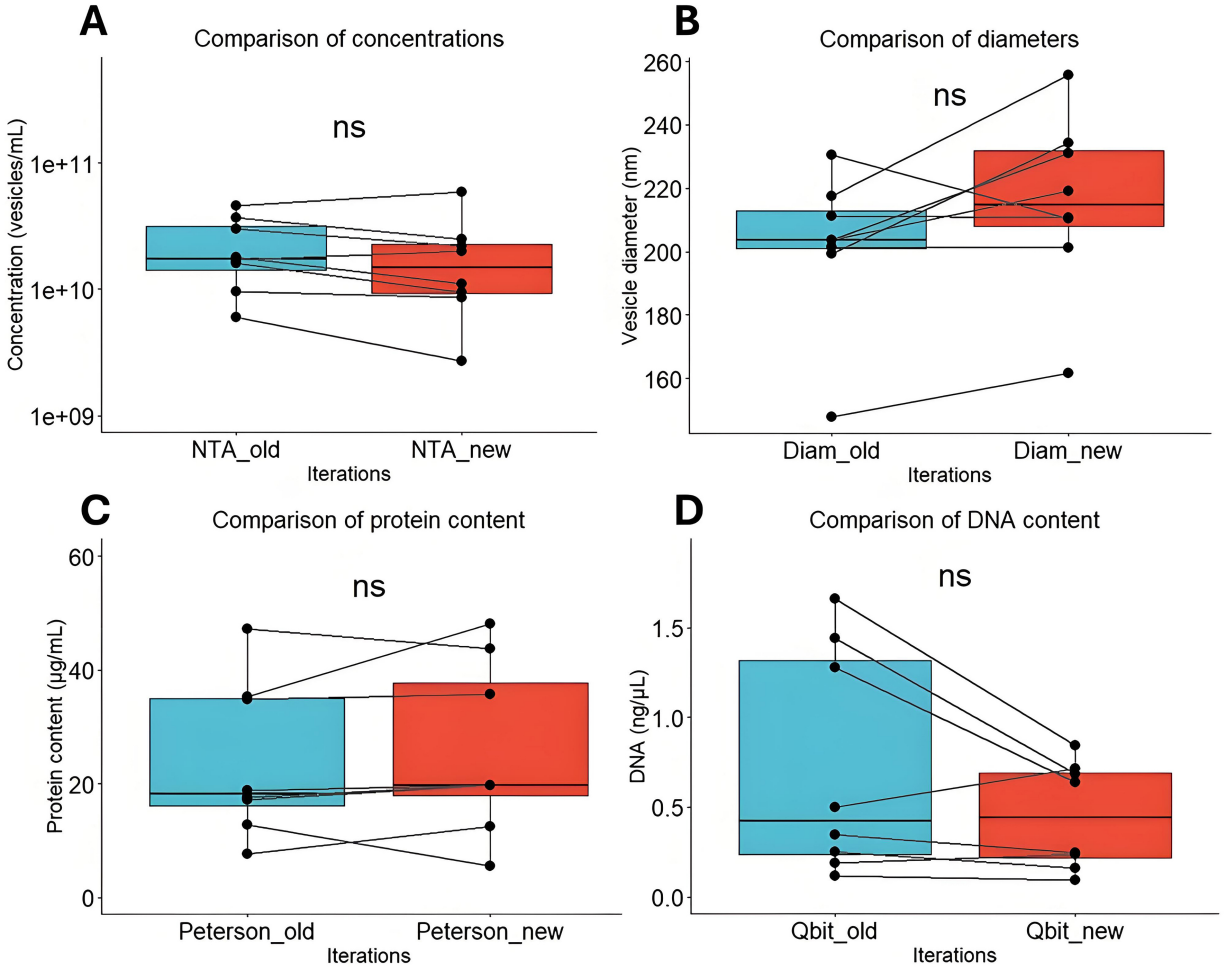
Comparison of bMV characteristics between iterations. (A) Particle counts (vesicles/mL) comparison between iterations; (B) Particle diameter comparison between iterations; (C) Vesicle protein quantification (μg/mL) between iterations; (D) Vesicle DNA quantification (ng/μL) between iterations. NS: Not significant; bMV: bacterial membrane vesicle; NTA: nanoparticle tracking analysis.

**Figure 4:**
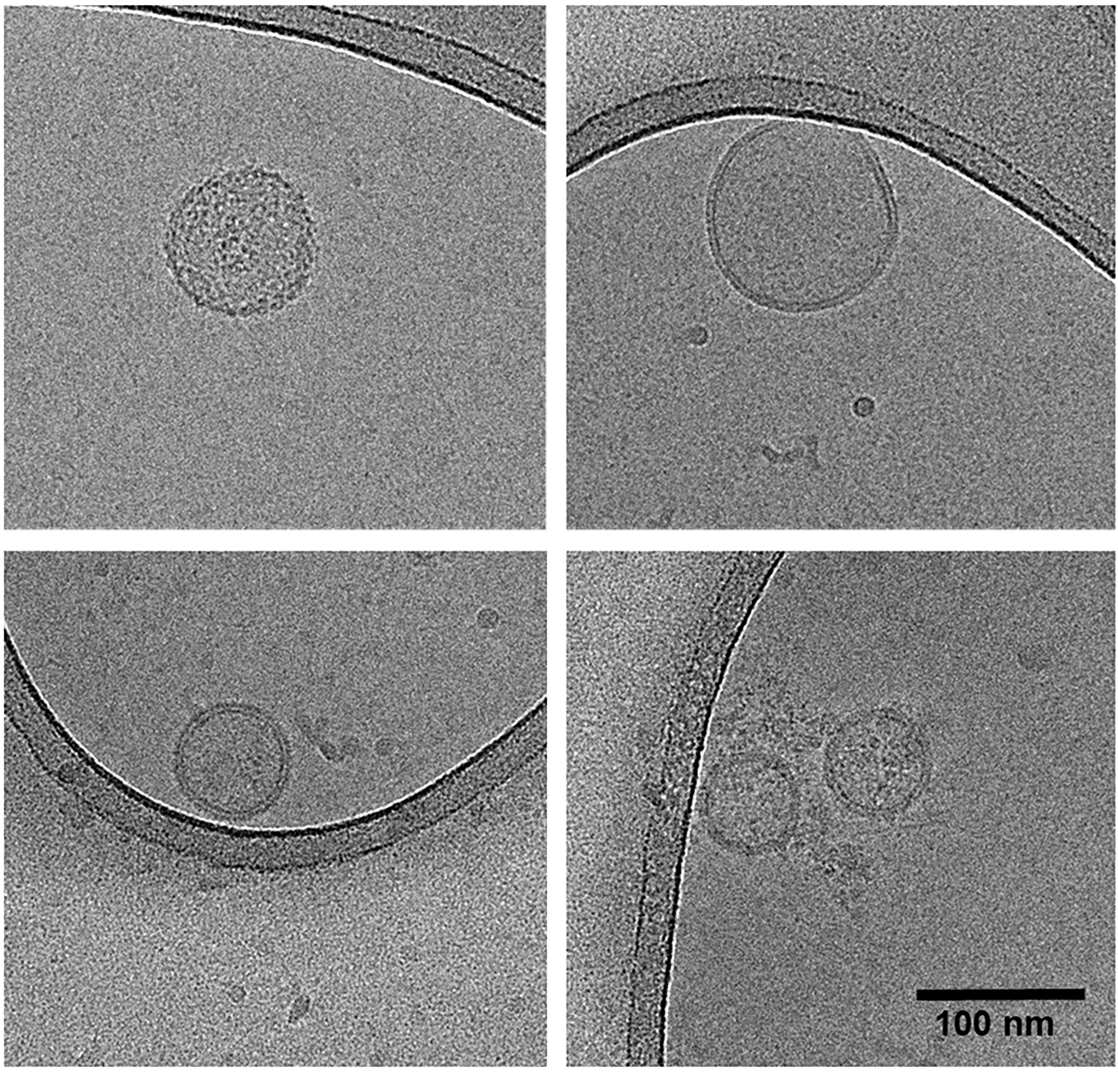
Cryo-transmission electron microscopy micrographs of feces-derived vesicles. Top left: Vesicle sample A1; top right: vesicle sample B1; bottom panels: vesicle sample C1. All samples originate from original sampling.

Between iterations, the mean vesicle yield in terms of particles per milliliter does not differ [Figure 3A], indicating the same number of vesicles can reliably be isolated from fecal samples following an additional freeze-thaw cycle. The herein obtained vesicles have the same mean diameter [Figure 3B], the same mean protein [Figure 3C], and DNA content [Figure 3D]. Although some variation is observed, sample metric rankings are generally conserved between iterations. Cryo-transmission electron microscopy (cryo-TEM) furthermore indicates that the spherical shape and intact membrane bilayer of the feces-derived membrane vesicles are retained following cryogenic storage and subsequent thawing prior to cryo-TEM sample preparation [Figure 4].

## Long-term storage does not affect bacterial compositions

**Figure 5:**
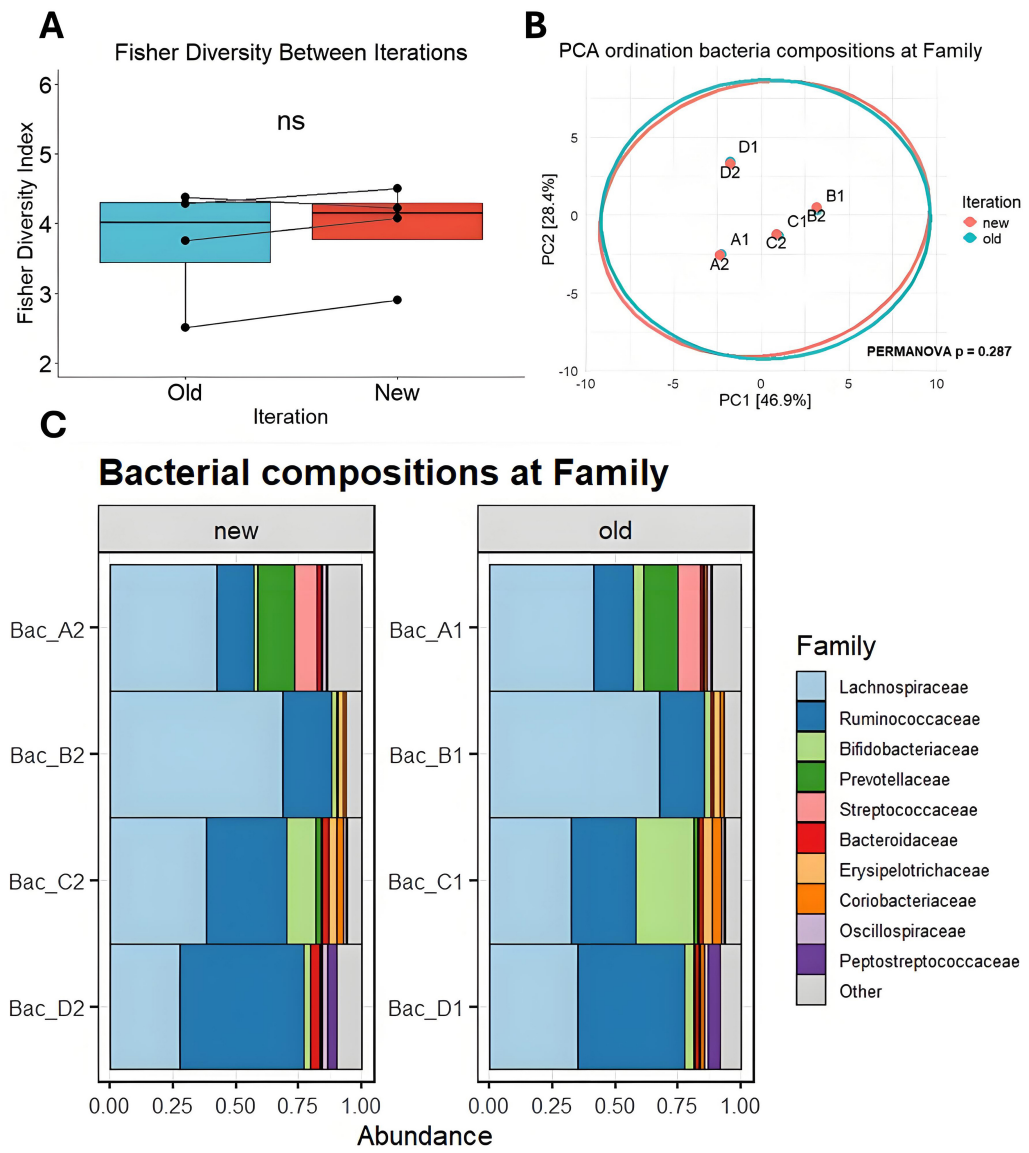
Comparison of bacterial composition across batch years. (A) Fisher’s alpha diversity at family taxonomy; (B) Principal component analysis ordination biplot of CLR-transformed features at the taxonomic Family level. 95% confidence ellipses are drawn per iteration; (C) Bar charts showing the proportional abundance of detected taxa from bacterial DNA at the Family level between iterations. PCA: Principal component analysis; CLR: centered log-ratio; NS: not significant; PERMANOVA: permutational multivariate analysis of variance.

Comparing the four fecal-derived bacterial samples between iterations revealed no significant differences in composition. Key bacterial taxa, including Lachnospiraceae and Ruminococcaceae, remained stably present at both timepoints for each of the four samples [Figure 5]. To assess potential differences in the number and distribution of bacterial taxa over time, alpha diversities were calculated. No significant differences in median diversity were observed between timepoints, suggesting that bacterial richness and evenness remained consistent [Figure 5A]. Principal component analysis (PCA) also indicates that the overall composition of each sample remains highly consistent over time [Figure 5B]. These findings indicate that fecal samples can be reliably stored at -80 °C for extended periods without significant alterations in bacterial composition [Figure 5C and Supplementary Figure 2].

## Vesicle fractions readily contain bacterial DNA

**Figure 6:**
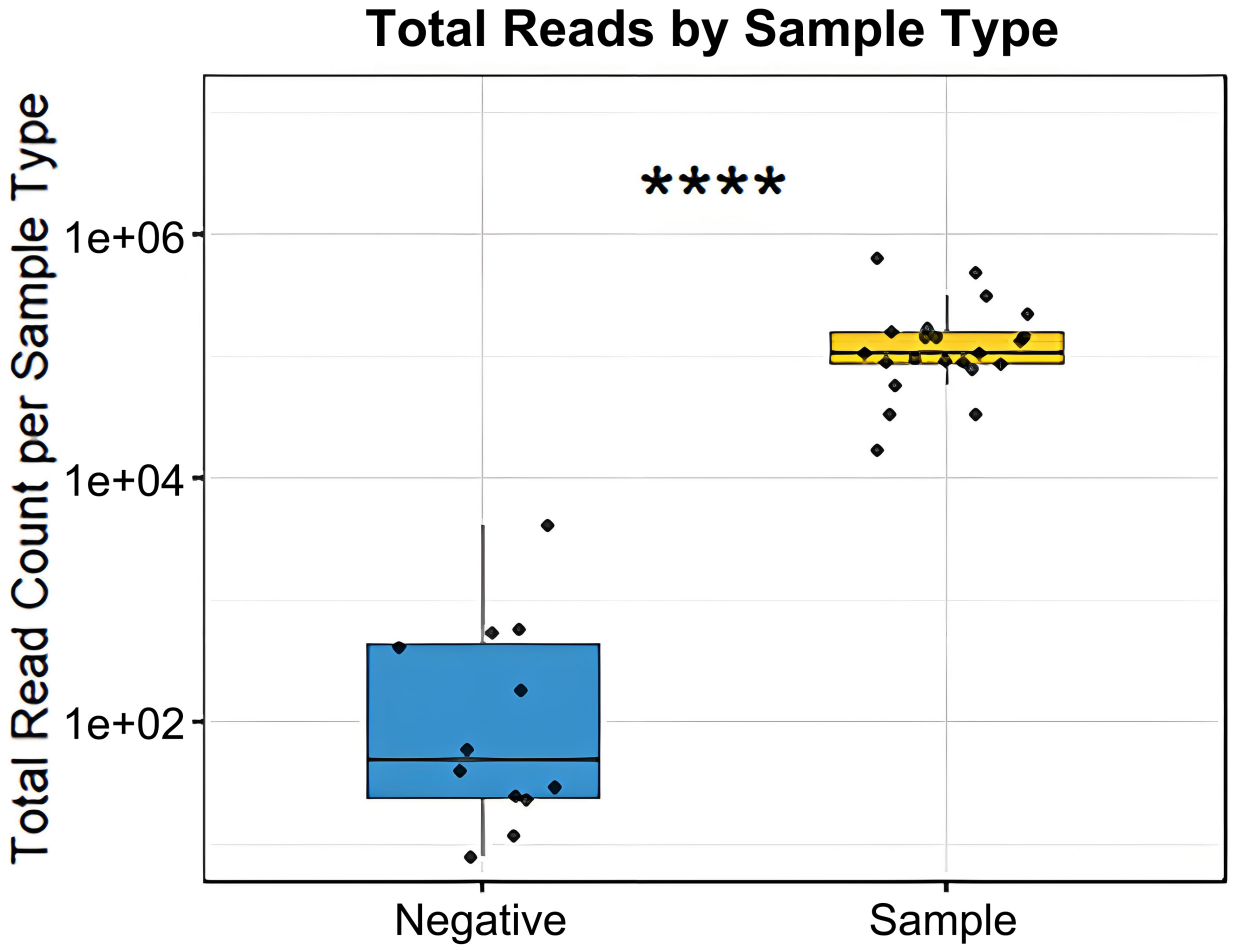
Comparison of 16S amplicon read counts between negative purification controls and vesicle samples (Wilcoxon rank-sum test, ^****^P < 0.0001).

Given that bMVs are of low biomass, we compared our vesicle-derived read counts to those of negative controls taken during the purification of bacteria and/or vesicles. Total read counts were significantly higher in vesicle samples compared to negative controls [Figure 6] following library amplification prior to Illumina sequencing.

## DISCUSSION

Reproducibility is an essential aspect of scientific research, constituting an important measure of reliability. Consistent results upon independent replication of experimental procedures strengthen credibility, demonstrating that observations are genuine and not dependent on artefacts or idiosyncrasies of experimental design. As interest in bMVs continues to increase - particularly microbiota-derived bMVs in gut-host interactions - the reuse of frozen fecal matter for bMV isolation is gaining prominence. To validate whether sample characteristics are preserved, we investigated the reproducibility of results obtained from the isolation and characterization of stored samples.

Comparing vesicle and bacterial compositions before and after extended storage times indicated no significant compositional differences, suggesting that the applied purification and analysis pipeline is reproducible, and that samples can be stably stored for reuse in later studies. The impact of long-term cryogenic storage on the reproducibility of fecal bacterial composition analysis has been previously investigated. For example, fecal samples stored at -80 °C for five years in RNA stabilization reagent exhibited high bacterial stability in terms of composition and diversity^[22]^. Furthermore, stool samples preserved at -70 °C retained their baseline compositions more effectively than those stored at -20 or 4 °C. Kim et al. found that room-temperature (20-25 °C) storage using a nucleic acid stabilizing agent outperformed cryogenic storage without stabilizing agents in preserving the original composition^[23]^. Others have reported that room-temperature storage of microbiome samples for 24 h, even without any excipients, introduced no significant bacterial compositional changes^[17,24]^.

Such studies illustrate the interest in proper sample storage conditions to ensure the validity of fecal microbiota analyses. However, to our knowledge, the stability of gut-bacterial vesicle DNA in fecal matter under various storage conditions has not yet been investigated thoroughly. Our analyses suggest that for a duration of > 6 months, storage at -80 °C is highly effective in preserving baseline composition without nucleic-acid stabilization agents. As reported before^[15]^, bacterial compositions differ from vesicle repertoires, as some bacterial taxa appear to be disproportionately efficient at producing vesicles. We observed this compositional difference when comparing vesicle reads [Figure 2] with bacterial reads [Figure 5].

Indeed, for optimal preservation of retained nucleic acids and proteins, storage of purified extracellular vesicles from both eukaryotic and prokaryotic sources is recommended at -80 °C, while repeated freeze-thaw cycles are best avoided^[16]^. Regarding bMVs, vesicles derived from *Neisseria meningitidis* retained structural integrity and immunogenicity when stored in aqueous matrices at -70 or 4 °C for a duration of one year. Fecal matter, however, is characterized by nuclease^[25]^ and protease^[26]^ activity originating from the vast enzymatic repertoire of the gut microbiota. In addition, host-derived lipases produced in the gastrointestinal tract^[27]^ could affect the membrane bilayer integrity of vesicles in fecal matter. Thus, vesicle 16S-rDNA could be subject to degradation by nucleases in fecal matter, as a consequence of membrane disruption by enzymatic action, freeze-thawing, or a combination thereof. If such a disruption of bMV membranes were to occur in a disproportionate manner (e.g., to a lesser extent for Gram-positive bacteria, whose bMVs can exhibit a protective peptidoglycan layer^[28,29]^), inadequate storage or unnecessary freeze-thawing could introduce profound compositional changes in the bMV repertoire. Encouragingly, the bMV repertoires we studied did not exhibit significant differences in composition or diversity between our experimental iterations.

Compared to negative controls, vesicle samples show markedly higher read counts, indicating a substantial vesicle-associated DNA load whose composition reflects the sample itself, rather than being solely driven by experimental artifacts or environmental contamination [Figure 6]. The relevance of negative control samples in marker gene sequencing arises from the use of template amplification (employing 35 cycles of PCR), in which potential contaminating DNA can profoundly affect compositions.

Nevertheless, a greater variability in taxa distribution was observed at the bMV level [Figure 2] compared to bacteria [Figure 5], suggesting that larger biomass bacterial samples can be more robustly assessed across timepoints. The observed stability of vesicle DNA is remarkable in light of the rich repertoire of fecal nucleases, proteases and lipases. This stability could be due to the physical encapsulation of vesicle DNA, given that the lipid bilayer is not permeable to charged molecules such as nucleases^[12,30]^. In addition, bacterial DNA could be surface-bound and associated or “entangled” with protein or peptidoglycan fragments, providing steric hindrance and reducing nuclease accessibility^[31]^. Steric hindrance mechanisms may also be involved in preserving structural integrity of the vesicle itself in the presence of lipases; as lipid bilayer can be shielded from enzymatic degradation by the presence of outer membrane proteins^[32]^, peptidoglycan^[31,33]^, lipotechoic acid^[34]^ or lipopolysaccharide (LPS)^[35]^.

Out of the 24 sequenced vesicle DNA runs (12 compositions at 2 timepoints), one sample exhibited a read count below the threshold for filtering, and only at the first timepoint. Intriguingly, this vesicle sample was found to contain 0.190 ng/μL of DNA, which was not the lowest concentration found [Figure 3D].

Limitations of the current study include its small sample size and the fact that samples were not investigated directly after collection. Additionally, given that study participants self-collected, (subtle) differences in exposure time to room-temperature prior to initial freezing, and consumer grade freezers not reaching -20 °C cannot be ruled out. Therefore, potential compositional or qualitative changes that may have occurred between sampling and cryogenic storage at our facility cannot be evaluated using the current study setup. Eventual effects of this and the effects of multiple (> 2) additional freeze thaw cycles on the compositional stability of bMV samples remain to be elucidated. It is especially important to note that samples of low-biomass are more prone to artefacts (e.g. contaminations, degradation). The aforementioned protease, nuclease and lipase activity in fecal matter^[25-27]^ might contribute to the degradation of vesicle-biomass. However, in the current study setup no investigation of the enzymatic degradation capacity in feces was performed. Assessment of this enzymatic degradation capacity in fecal samples could potentially help explain why some samples exhibited greater stability over time than others.

Nevertheless, compositions of microbiome samples (bacteria) were previously found to be robust against changes induced by (initial) freeze-thawing^[17,24]^. Such characterizations have not been performed for bMV DNA compositions to date. Lastly, as some methods of vesicle characterization were not bMV-specific, it is possible that a minority^[15]^ of the vesicles in a feces-derived vesicle repertoire may be of host-cell origin. Lastly, 16S rRNA marker gene sequencing does not generally facilitate accurate below-genus level identification of microbial taxa. It therefore remains to be investigated if compositional stability persists at species- and strain level taxonomy.

In conclusion, we investigated the reproducibility of the isolation and characterization of gut-bacteria and their bMVs using marker-gene sequencing, using a small but representative human-derived sample set. Our results confirm that fecal samples intended for bMV isolation can be stably stored at -80 °C over extended periods, with negligible effects on composition and/or physicochemical characteristics induced by freeze-thawing. Follow-up studies would benefit from investigating bMV samples before and after freezing, as well as from including more sophisticated controls, employing nucleases to investigate vesicle cargo rigidity.

As gut-bacteria-derived membrane vesicles attract growing interest for their versatile biological properties and ability to influence the human host, it is recommended to study them alongside the feces-derived microbes that produced them. It is imperative that sample characteristics remain stable during storage, as this will permit the retrospective analysis of bMV repertoires in cryogenically preserved fecal samples. Our findings are relevant for bMV research as they provide a foundation for future studies leveraging existing sample collections to explore roles of these vesicles in health and disease.

## DECLARATIONS

### Availability of data and materials

Sequencing data has been deposited to the European Nucleotide Archive (PRJEB85803).

### Copyright

© The Author(s) 2025.
