# Supplementary material for: Assessment of the reproducibility of bacterial membrane vesicle isolation and characterization
Source: Extracell Vesicles Circ Nucl Acids. 2025 Nov 10;6(4):728–41. doi: 10.20517/evcna.2025.71 (PMC12809393; doi:10.20517/evcna.2025.71)
Supplement: Supplementary file 1 [file evcna-6-4-728-SupplementaryMaterials.pdf]

Supplementary Materials

Assessment of the reproducibility of bacterial membrane vesicle isolation and characterization

Jari Verbunt[1,2], Johan Jocken[2], Emanuel Canfora[2], David Barnett[1], Ellen E. Blaak[2], Paul Savelkoul[1], Frank Stassen[1]

[1]Department of Medical Microbiology, Infectious Diseases & Infection Prevention, School of Nutrition and Translational Research in Metabolism (NUTRIM), Maastricht University Medical Center+, Maastricht 6202 AZ, The Netherlands.
[2]Department of Human Biology, School of Nutrition and Translational Research in Metabolism (NUTRIM), Maastricht University Medical Center+, Maastricht 6202 AZ, The Netherlands.

Correspondence to: Dr. Frank Stassen, Department of Medical Microbiology, Infectious Diseases & Infection Prevention, School of Nutrition and Translational Research in Metabolism (NUTRIM), Maastricht University Medical Center+, Maastricht 6202 AZ, The Netherlands. E-mail: F.Stassen@maastrichtuniversity.nl

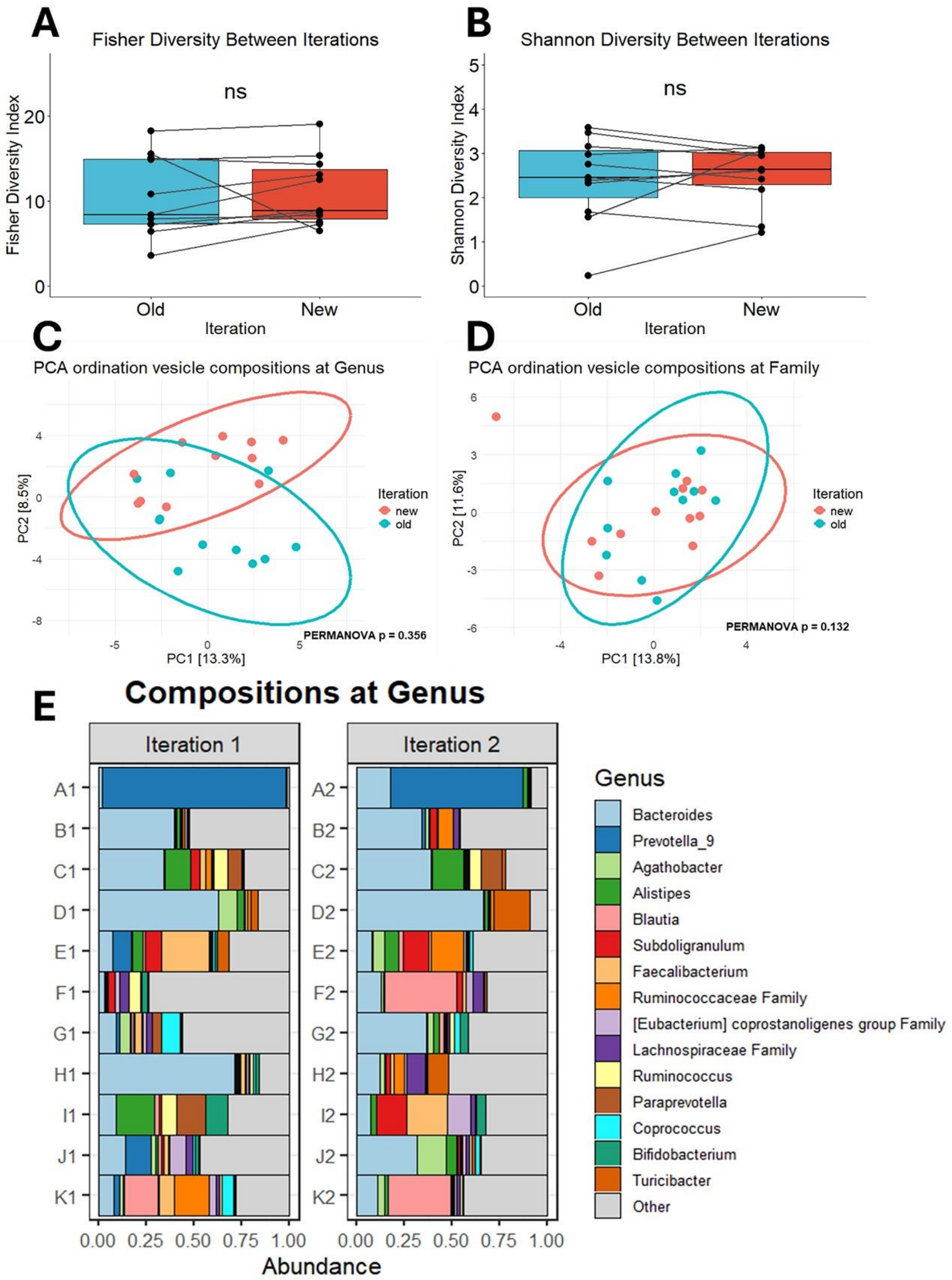

Supplementary Figure 1. Comparison of bMV compositions between iterations. (A) Fisher's alpha diversity at the family taxonomy level. Data are presented as means ± SEM; (B) Principal component analysis ordination biplot of CLR-transformed features at the taxonomic genus level. 95% confidence ellipses are drawn per iteration; (C) Bar charts showing the proportional abundance of detected taxa from vesicle DNA at the genus level between iterations. NS: Not significant.

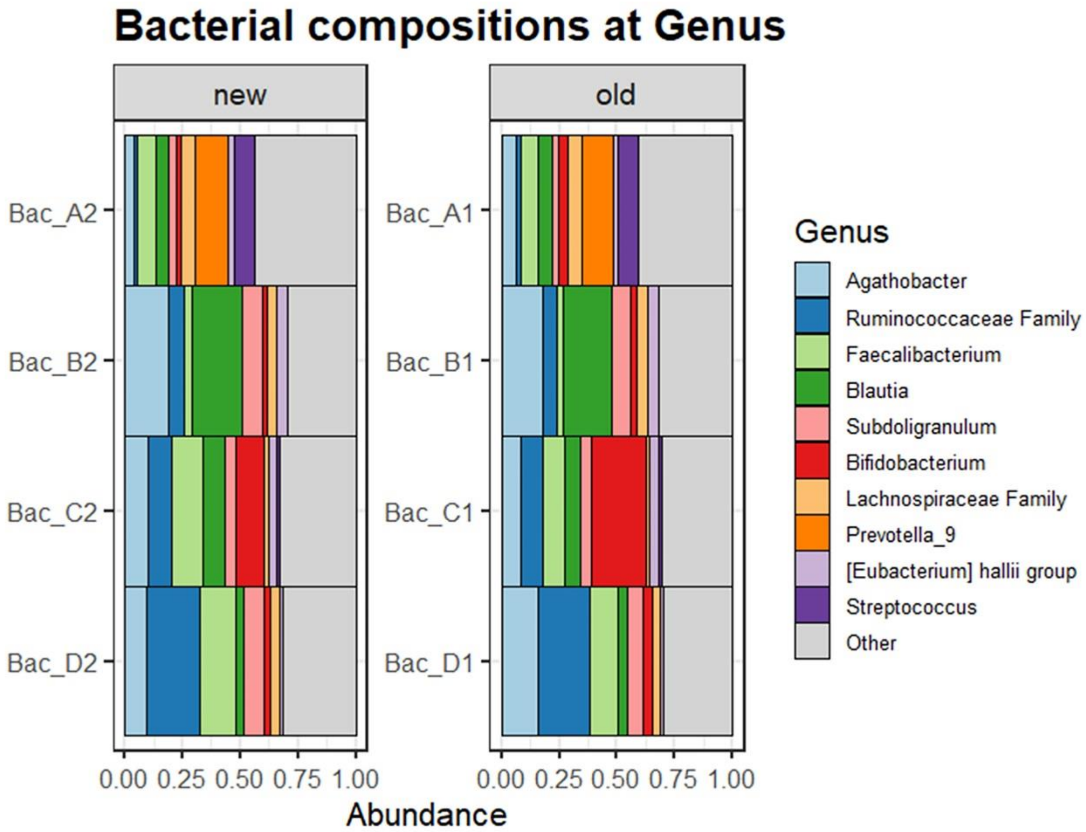

Supplementary Figure 2. Bar charts showing the proportional abundance of detected taxa from bacterial DNA at the genus level between iterations.
